# Prevalence and distribution of *Eimeria* species in broiler chicken farms of different capacities

**DOI:** 10.1051/parasite/2013052

**Published:** 2013-12-06

**Authors:** Adriana Györke, Loredana Pop, Vasile Cozma

**Affiliations:** 1 Parasitology and Parasitic Diseases Department, Faculty of Veterinary Medicine, University of Agricultural Science and Veterinary Medicine Cluj Napoca 3-5 Calea Mănăştur 400372 Cluj-Napoca Romania

**Keywords:** *Eimeria*, Prevalence, Chicken, PCR, Romania

## Abstract

We conducted a survey in broiler farms from Romania to establish prevalence and distribution of *Eimeria* species using single PCR assay. We found *Eimeria* spp. in 21 (91%) out of 23 flocks, and in 11 (92%) out of 12 farms. Four species of *Eimeria* were identified: *E. acervulina* (21/23; 91%), *E. tenella* (14/23; 61%), *E. maxima* (5/23; 22%) and *E. praecox* (3/23; 13%). Infection with a single species (*E. acervulina*) was detected in 6 (26%) infected flocks originated from large farms. Mixed infections were found in 15 (65%) flocks and the most prevalent combination was *E. acervulina* + *E. tenella* (8/23; 35%). Four flocks (17%) harboured mixed infection with *E. acervulina + E. tenella* + *E. maxima*. *E. acervulina* was significantly more prevalent in flocks that received ionophores as anticoccidial feed additives.

## Abstract (fr)

Nous avons mené une enquête dans les élevages de poulets de chair en Roumanie pour établir la prévalence et la répartition des espèces d’*Eimeria* en utilisant les tests PCR. Nous avons trouvé *Eimeria* spp. chez 21 (91 %) des 23 bandes de poules, et dans 11 (92 %) des 12 fermes. Quatre espèces d’*Eimeria* ont été identifiées : *E. acervulina* (21/23 ; 91 %), *E. tenella* (14/23 ; 61 %), *E. maxima* (5/23 ; 22 %) et *E. praecox* (3/23 ; 13 %). L’infection par une seule espèce (*E. acervulina*) a été détectée dans 6 (26 %) des bandes infectées provenant de grandes exploitations. Des infections mixtes ont été trouvées dans 15 (65 %) des bandes et la combinaison la plus fréquente était *E. acervulina* + *E. tenella* (8/23 ; 35 %). Quatre bandes (17 %) hébergeaient une infection mixte à *E. acervulina* + *E. tenella* + *E. maxima*. *E. acervulina* était significativement plus fréquente chez les bandes recevant des ionophores comme suppléments alimentaires anticoccidiens.

## Introduction

Coccidiosis is one of the most important and costly diseases of poultry industry worldwide. The aetiological agents are apicomplexan protozoan parasites from *Eimeria* genus that multiply in the epithelial cells of the intestine. In poultry, there are seven recognized species that develop in certain parts of the gut (site-specific), each causing a separately recognizable disease [56]: *E. acervulina, E. tenella, E. maxima, E. necatrix, E. mitis, E. praecox* and *E. brunetti.* These species of *Eimeria* have different pathogenicity; *E. tenella* and *E. necatrix* are the most pathogenic and cause bloody lesions, high morbidity and mortality in naive chickens [19, 32]; *E. acervulina*, *E. maxima* and *E. brunetti* also cause clinical diseases; *E. praecox* and *E. mitis*, although considered to be relatively non-pathogenic [32], do cause a reduced feed conversion efficiency and growth rate [56]. Also, infection with certain species of *Eimeria* was demonstrated to be implicated in predisposing birds to necrotic enteritis [55], through lesions that compromise gut integrity, and allow the proliferation of pathogens [52].

Intensive chicken farming depends on specific prophylaxis of coccidiosis with in-feed anticoccidial drugs and live vaccines. Over time, the coccidiostats have become less effective due to development of drug resistance. Drug-resistant *Eimeria* strains are responsible for subclinical coccidiosis and, subsequently, for impaired economical performance as body weight gain, and feed conversion ratio [44]. The economic losses are significant, being estimated at more than 3 billion US$ annually in the world [11], and the economic importance of subclinical coccidiosis varies with composition of coccidial populations [16]. Therefore, identification and genetic characterization of different species of *Eimeria* are central to prevention, surveillance and control of coccidiosis [31].

Identification of *Eimeria* species is based on clinical features, specific lesions in certain sites of the intestine, and morphological and biological features as sizes of oocysts, sites of infection, pre-patent period, sporulation time. Although, *E. maxima* can be easily identified based on oocyst size, while *E. tenella* and *E. necatrix* produce unmistakable lesions [14], identification through these parameters only is not always accurate due to overlapping characteristics [27]. Mixed infections are commonly found under field conditions, which pose a problem for the precise discrimination of species using classical methods. Moreover, classical methods are expensive, time-consuming [17] and require highly trained personnel [27].

Polymerase chain reaction (PCR) based assays proved to be effective for identification of all seven species of *Eimeria* in chickens. The used target regions are small subunit rRNA [48], 5S rRNA [46], first and second internal transcribed spacers (ITS-1; ITS-2) of nuclear ribosomal DNA [13, 15, 40], and sequence-characterized amplified region (SCAR) derived from random amplified polymorphic DNA (RAPD) profiles [12].

We conducted a survey in broiler chicken farms in Romania from August to November 2010. The aim was to establish the prevalence and distribution of *Eimeria* species by PCR in different size broiler farms with different prophylactic programmes.

## Material and methods

### Broiler industry in Romania

Union of poultry breeders from Romania has 276 members, of which 18 large poultry companies that produce over 10 thousand tons of meat/year/farm, 22 medium poultry companies that produce between 5 and 10 thousands tons of meat/year/farm and 236 small poultry companies that produce less than 5 thousands tons of meat/year/farm [49]. The production of poultry meat in 2010 was about 317 thousands tons. Average performances in the same year were: daily body weight gain 54.19 g; feed conversion ratio of 1.859; mortality of 4.24%; and European Production Index 299.15 [49].

The most common broiler breeds are Cobb500 and Ross308, and they are reared in houses made of concrete on wood shavings. Prophylaxis of coccidiosis is based on the use of in-feed anticoccidial drugs.

The average age at slaughter is about 42 days, and average live weight of 2.2 kg. The time between successive grow-outs is about 2–3 weeks. Used litter is removed and the broiler houses are cleaned and chemically disinfected.

### Study flocks and samples

The study was conducted in 12 broiler farms from Romania picked by simple random sample, during August–November 2010. Farms were subsequently divided according to their size in three groups: small (*n* = 4), medium (*n* = 3) and large (*n* = 5) farms. Prophylaxis of coccidiosis was made with different ionophores and chemicals as it is stated in Table 1.

**Table 1.** Species-specific primers targeting the ITS-1 region for *Eimeria* species that infect chickens*

Species	Primer sequence 5′ 3′	Annealing temperature (°C)	Amplicon size (bp)
*E. acervulina*	F 5′-GGGCTTGGATGATGTTTGCTG-3′	65	145
	R 5′-GCAATGATGCTTGCACAGTCAGG-3′		
*E. brunetti*	F 5′-CTGGGGCTGCAGCGACAGGG-3′	58	183
	R 5′-ATCGATGGCCCCATCCCGCAT-3′		
*E. maxima*	F 5′-GTGGGACTGTGGTGATGGGG-3′	65	205
	R 5′-ACCAGCATGCGCTCACAACCC-3′		
*E. mitis*	F 5′-GTTTATTTCCTGTCGTCGTCTCGC-3′	65	330
	R 5′-GTATGCAAGAGAGAATCGGGATTCC-3′		
*E. necatrix*	F 5′-AGTATGGGCGTGAGCATGGAG-3′	58	160
	R 5′-GATCAGTCTCATCATAATTCTCGCG-3′		
*E. praecox*	F 5′-CATCGGAATGGCTTTTTGAAAGCG-3′	65	215
	R 5′-GCATGCGCTAACAACTCCCCTT-3′		
*E. tenella*	F 5′-AATTTAGTCCATCGCAACCCTTG-′	65	278
	R 5′-CGAGCGCTCTGCATACGACA-3′		

* Primers previously described by Haug et al. [15] and Schnitzler et al. [40, 41].

We collected faeces samples from 2 flocks/farm, except farm “D”, in total 23 flocks, when chickens were 20–35 days old (median 28 days), and information regarding coccidiostat drugs used (Table 2). Approximately 250 g of fresh faecal droppings/sample was collected at random by hand along the feed and water lines. The samples were processed once they arrived in the laboratory by flotation method with saturated sodium chloride (specific gravity 1.18–1.2) and stored at 4 °C till the next day. Afterwards, oocysts were isolated, purified and concentrated from faeces with saturated salt solution [43] and sporulated in 2.5% potassium dichromate solution. The oocysts were washed free from the salt and potassium dichromate by repeated centrifugation and resuspended in tap water. At the end, molecular analysis was done by PCR in order to identify the species of *Eimeria.*

**Table 2.** List of field samples and the results of ITS-1 PCR.

Farm	Flock	Age (days)	Coccidiostat^a^	Total	*E. acervulina*	*E. tenella*	*E. maxima*	*E. praecox*	Single infection	Mixed infections
Small-size farms (8 flocks)										
A	1	28	Lasalocid (Avatec)	+	+	+	–	–	–	+(A + T)
A	2	32	Lasalocid (Avatec)	+	+	+	–	–	–	+(A + T)
F	1	30	Lasalocid (Avatec)	+	+	+	–	+	–	+(A + T+P)
F	2	26	Lasalocid (Avatec)	+	+	+	–	–	–	+(A + T)
K	1	25	Maduramycin (Cygro)	+	+	+	–	–	–	+(A + T)
K	2	25	Maduramycin (Cygro)	+	+	+	–	–	–	+(A + T)
L	1	28	nd	+	+	+	+	–	–	+(A + T+M)
L	2	25	nd	+	+	+	+	+	–	+(A + T+M + P)
Total *n*(%)		Med. = 27		8(100)	8(100)	8(100)**	2(25)	2(25)	0	8(100)*
Medium-size farms (6 flocks)										
B	1	29	Narasin + nicarbazin (Maxiban)	+	+	+	–	–	–	+(A + T)
B	2	29	Narasin + nicarbazin (Maxiban)	+	+	+	+	–	–	+(A + T+M)
C	1	20	Diclazuril (Clinacox)	–	–	–	–	–	–	–
C	2	32	Diclazuril (Clinacox)	–	–	–	–	–	–	–
G	1	29	Monensin (Coxidin)	+	+	+	–	–	–	+(A + T)
G	2	29	Monensin (Coxidin)	+	+	+	–	–	–	+(A + T)
Total *n*(%)		Med. = 29		4(66,7)	4(66,7)	4(66,7)	1(16,7)	0	0	4(66,7)
Large-size farms (9 flocks)										
D	1	nd	Robenidine(Cycostat)	+	+	–	–	–	+(A)	–
E	1	28	Salinomycin (Sacox)	+	+	–	–	–	+(A)	–
E	2	27	Salinomycin (Sacox)	+	+	–	–	–	+(A)	–
H	1	28	Diclazuril (Clinacox)	+	+	–	–	+	–	+(A + P)
H	2	35	Diclazuril (Clinacox)	+	+	–	–	–	+(A)	–
I	1	28	nd	+	+	–	–	–	+(A)	–
I	2	28	nd	+	+	–	–	–	+(A)	–
J	1	35	Monensin (Coxidin)	+	+	+	+	–	–	+(A + T+M)
J	2	28	Monensin (Coxidin)	+	+	+	+	–	–	+(A + T+M)
Total *n*(%)		Med. = 28		9(100.0)	9(100.0)	2(22.2)	2(22.2)	1(11.1)	6(75.0)**	3(33.3)
Total *n*(%)	*n* = 23	Med. = 28		21(91,3)	21(91.3)***	14(60,9)	5(21,7)	3(13,0)	6(26.1)	15(65,2)**
Ionophores (*n =* 14)				14(100)*	14(100)*	12(85.7)**	3(21.4)	1(7.1)	2(14.3)	12(85.7)*
Chemicals (*n* = 5)				3(60)	3(60)	0	0	1(20.0)	2(40.0)	1(20.0)

a Nineteen out of 23 farmers answered to question regarding the coccidiostat used in-feed for coccidiosis control. A = *E. acervulina;* T = *E. tenella*; M = *E. maxima*; P = *E. praecox*. + positive; − negative. Fisher exact test: *p < 0.05; **p < 0.01; ***p < 0.001. nd = not done; Med. = median.

### DNA extraction

DNA extraction from sporulated oocysts of each flock sample was performed with the commercial kit Isolate Fecal DNA Kit (Bioline; Cat. No. BIO-52038). We followed the manufacturer’s instructions with minor modifications; we used 200 μL of sporulated oocysts suspension, instead of 150 mg faeces and the grinding time was 10 min instead of 1 min. The kit contains for the first step of extraction tubes with beads (bashing bead lyses tube). The DNA was stored at −20 °C till using.

### Polymerase chain reaction (PCR)

*Eimeria* species were identified by single PCR assay using species-specific primers (Table 1) targeting the internal transcribed spacer-1 (ITS-1) as previously described by Schnitzler et al. [40, 41] and Haug et al. [15]. Each reaction mixture of 25 μL contained: 2 μL DNA sample; 25 pmol of species-specific reverse and forward primers; 12.5 μL MyTaq™ Mix (Bioline, Cat. No. BIO-25041); 9 μL ultra-pure water (PCR Water, Cat. No. BIO-37080, Bioline); and 0.5 μL of 1% bovine serum albumin. We used Houghton strains of all seven *Eimeria* species that infect chickens obtained from VLA (Veterinary Laboratory Agency Weybridge, UK) as positive controls, and distilled water as negative control.

The amplification was performed in MyGenie™ 96 Gradient Thermal Block (Bioneer). The cycling parameters for the amplification were the following: an initial denaturation at 95 °C for 1 min, followed by 35 cycles of denaturation (95 °C, 15 s), annealing (58 or 65 °C, 15 s) and extension (72 °C, 10 s), with a final extension at 72 °C for 3 min.

The PCR products (8 μL), mixed with loading buffer (2 μL), were separated on a 1.5% agarose gel by electrophoresis, stained with SYBR® Green I Nucleic Acid Gel Stain (Invitrogen). Specific fragments were identified by size using a 100 bp ladder under UV light (BioDoc-It® Imaging Systems, UVP®, VWR International LLC).

### Statistical analysis

Data were statistically analysed with Epi Info version 3.5.2 [10]. First, the frequency and prevalence of species detected and the species combinations were recorded as overall, by farm type (small, medium and large farms) and type of coccidiostat used (ionophores, chemicals). Then, the difference in the prevalence was evaluated using Fisher exact test. A *p* value of < 0.05 was statistically significant.

## Results

**Figure 1:**
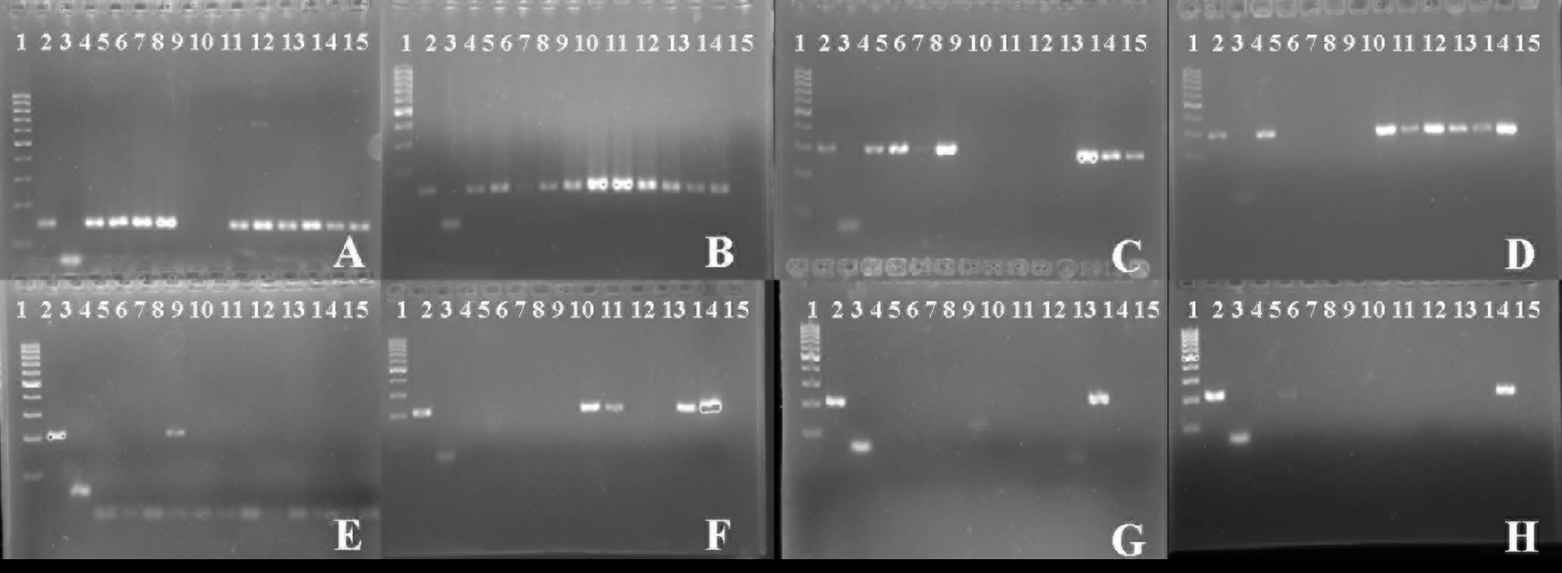
Results obtained in PCR following agarose gel electrophoresis. Lines: L1 100 bp ladder; L2 positive control; L3 negative control; L 4–15 samples. (A, B) *E. acervulina* 145 bp; (C, D) *E. tenella* 278 bp; (E, F) *E. maxima* 205 bp; (G, H) *E. praecox* 215 bp.

We found *Eimeria* spp. by PCR in 21 (91%) out of 23 flocks, and in 11 (92%) out of 12 farms. Four species of *Eimeria* were identified: *E. acervulina, E. tenella, E. maxima* and *E. praecox* (Figure 1). Overall, the most prevalent species was *E. acervulina* (21/23; 91%), followed by *E. tenella* (14/23; 61%) (Table 2). *E. acervulina* was overall significantly more prevalent in flocks with in-feed ionophores (*p* < 0.05). The overall prevalence of *E. tenella* was statistic significantly higher in small farms (8/8; 100%; *p* < 0.05), and in flocks that received ionophores as anticoccidial feed additives (*p* < 0.01).

Infection with only one species (*E. acervulina*) was detected in six (26%) positive flocks; these flocks originated from large farms (Table 2). Mixed infections with two or more species were found in 15 (65%) flocks (Table 2); the prevalence of mixed infections was significantly higher (*p* < 0.05) in small farms (8/8; 100%), and in the farms where chickens received ionophores as anticoccidial feed additives (12/14; 86%; *p* < 0.05), than in medium (4/6; 67%) or large farms (3/9; 33%), and those that received chemicals as anticoccidial feed additives (1/5; 20%).

The most prevalent combinations were *E. acervulina* + *E. tenella* (8/23; 35%; *p* < 0.05), and significantly more prevalent in small farms (5/8; *p* < 0.05) and in farms using ionophores (8/14; 57%; *p* < 0.05) (Table 3). Four flocks (17%) harboured mixed infection with *E. acervulina + E. tenella* + *E. maxima*. Other infection combinations found were: *E. acervulina* + *E. praecox*; *E. acervulina* + *E. tenella* + *E. praecox;* and *E. acervulina* + *E. tenella* + *E. maxima* + *E. praecox.*

**Table 3:** Frequency and prevalence [*n*(%)] of mixed infections in broiler farms from Romania.

	Total (*n* = 23)	Farm size			Coccidiostat^a^	
		Small (*n* = 8)	Medium (*n* = 6)	Large (*n* = 9)	Ionophores (*n* = 14)	Chemicals (*n* = 5)
*E. acervulina + E. tenella*	8(34,8)*	5(62,5)*	3(50,0)	0	8(57,1)*	0
*E. acervulina + E. praecox*	1(4,3)	0	0	1(11,1)	0	1(20,0)
*E. acervulina + E. tenella + E. maxima*	4(17,4)	1(12,5)	1(16,7)	2(22,2)	3(21,4)	0
*E. acervulina + E. tenella + E. praecox*	1(4,3)	1(12,5)	0	0	1(7,1)	0
*E. acervulina + E. tenella + E. maxima +E. praecox*	1(4,3)	1(12,5)	0	0	0	0

Fisher exact test: *p < 0.05; **p < 0.01; ***p < 0.001.

a Nineteen out of 23 farmers answered to question regarding the coccidiostat used in-feed for coccidiosis control.

## Discussion

Epidemiological studies on the prevalence of *Eimeria* species are useful tools for prevention and control of coccidiosis [31, 34]. Also, PCR-based assays can identify with accuracy species of *Eimeria* that afflict animals at farm level, even when they harbour mixed infections with a relative frequency down to 0.05% (two oocysts per PCR/4000) [17].

We identified species of *Eimeria* by PCR in 91% (21/23) of samples, although by flotation oocysts have been seen in all samples (23/23). In negative flocks to PCR there were few oocysts per gram faeces, between one and 117 (data not shown). Detection level by PCR is around 0.4–50 oocysts and depends mainly on sensitivity of the protocol used for DNA extraction [15]. Most of the protocols use glass beads in the first step of DNA extraction, and bead sizes and grinding times may influence the amount of DNA recovered from the sample [15]. Another cause can be the low number of oocysts per gram faeces in the samples. Haug et al. [15] found that when oocyst concentration is low, oocyst grinding is less efficient in extracting sample DNA.

We found that broiler chicken farms in Romania are populated with four species of *Eimeria: E. acervulina, E. tenella, E. maxima* and *E. praecox*. The most prevalent species were *E. acervulina* (91%) and *E. tenella* (61%). The crowding effect [51] and interactions among *Eimeria* species [50] are the most important factors affecting oocysts production. *E. acervulina* and *E. tenella* have the highest reproductive potential [51], and in mixed infections, *E. acervulina* reduces the oocysts production of *E. brunetti, E. maxima, E. tenella* and *E. necatrix*. We can suspect the same effect against *E. praecox,* because they occupy the same part of the gut. Besides, the crowding effect seems to be modulated by availability of epithelial cells and immunogenicity [51]. Immune response, developed after a primary infection, reduces the number of oocysts, and depends on immunogenicity of each *Eimeria* species. It is well known that *E. maxima* is the most immunogenic species in chickens, and *E. acervulina* and *E. tenella* have a moderate to low immunogenicity [29, 38]. Also, anticoccidial drugs interfere with the development of immunity in chickens. Broadly speaking, ionophores in low concentration stimulate the immune response, and in higher doses have immunosuppressive effect [33, 42]. Long et al. [28] found that monensin at 60–100 ppm reduced the immune response to infection with *Eimeria*, while concentration of 40 and 50 ppm allowed good development of immunity. Another ionophore, lasalocid, interferes partially with the development of immunity against *E. tenella* [39]. As regards chemicals, it was concluded that under experimental condition diclazuril did not significantly interfere with protective immunity formation against *E. tenella* [30]. As a conclusion, Hu et al. [18] found in a study using monensin, narasin, lasalocid, salinomycin, nicarbazin, halofuginone, robenidine, and amprolium and field isolates of *Eimeria acervulina*, *E. maxima* and *E. tenella* that none of the drugs interfered appreciably with protective immunity against *Eimeria*. Otherwise protection against infection with *Eimeria* is acquired gradually and it is complete at 7 weeks of age [7].

Haug et al. [16] found that long-term use of narasin between 2001 and 2004 conducted to a shift of coccidial population from a dominance of medium and large oocysts represented by *E. tenella, E. praecox* and *E. maxima* to a dominance of small oocysts as *E. acervulina* and an increase in flock prevalence. It is well known that long-term use of anticoccidials leads to development of drug resistance [6]. Drug resistance to anticoccidial drugs is described worldwide to all coccidiostats and to all *Eimeria* species [2, 21, 35, 36, 45, 53, 57]. Generally, *E. acervulina* seems to have a faster rate of drug resistance development and consequently a wide spectrum of resistance explained by its high reproductive index and the short life cycle [5, 20]. This can be an explanation for preponderance of *E. acervulina* in our study, or even in others.

In Czechoslovakia, France and Sweden during 1990–1996, all seven species of *Eimeria,* occurring in mixed infections, were reported from broiler farms [23, 54]. After 2000, in Norway, *E. acervulina* (100%), *E. tenella* (77%) and *E. maxim*a (25%) were the predominant species as we report in our study, including low percentages of *E. praecox* (10%) and *E. necatrix* (2%) [17]. In North America, (Ontario, Canada), Ogedengbe et al. [34] found the same species as in European countries, but with a significantly lower prevalence between 0.3 and 2.5%. Also, in a study with chickens raised on used litter in the USA, the predominant species were *E. acervulina*, *E. maxima*, *E. praecox* and *E. tenella*, according to Lee et al. [25]. In Africa, Middle East and Asia the most frequent species reported in broilers are *E. brunetti* (between 10 and 60%) and *E. necatrix* (4–30%) [1, 4, 24, 26, 44]. In the same geographical areas, *E. tenella* was the most prevalent species [4, 24], except in Iran where as in Europe, Australia and North America the most prevalent species was *E. acervulina* [44].

In the present study *E. tenella* was the second species found as the most prevalent overall, presenting the same statistical level (*p <* 0.01) of infection as *E. acervulina* in small and medium farms. However, *E. tenella* was less prevalent in large farms, and some authors reported that the flock size did not affect the prevalence [3]. *E. tenella* is one of the most pathogenic species. It causes caecal lesions as haemorrhages, oedema, necrosis and anaemia [19]. Moreover, in an experiment it was observed that *E. tenella* infection can be a cause of the recrudescence of *Salmonella enteritidis* [37].

Multiple infections with different species of *Eimeria* in chickens are a common situation in most of the farms [1, 17, 54]. We found mixed infection (2–4 species) in 65% of the cases and single infection (*E. acervulina*) in 22% of the cases. Single infection was observed only in large farms (75%) and was more prevalent than mixed infections (38%) in these farms. Haug et al. [17] found single infection with *E. acervulina* (16%) less prevalent in Norway than in our study in Romania. Mixed infections were associated with small and medium farms and with ionophores. The same findings were observed in China, Shandong province, in small-scale farms where more than one *Eimeria* species existed in most of the samples [47]. Most likely, the mixed infections are more prevalent in small and medium farms due to poor management and biosecurity practises as high stocking densities, reduced time between successive grow-outs [26], microclimate and workers [22]. As regarding ionophores (alter ion transport and disrupt osmotic balance), they do not prevent replication of *Eimeria* completely [9] as chemicals do (affect parasite metabolism). In order to prevent drug resistance, rotation of coccidiostats and shuttle programmes are recommended. Nevertheless, drug resistance is widespread and it was described to all coccidiostats and *Eimeria* species [8, 35]. Small and medium farms in Romania do not have their own feed mill and in most cases they cannot control the prophylaxis programme (personal observation).

The high prevalence of infection with *E. acervulina* and *E. tenella* as single or multiple infections in Romanian broiler farms can be due to reduced susceptibility to anticoccidial drugs and to poor management practises, especially in small and medium farms. Further investigations are needed in order to determine the susceptibility of these strains to coccidiostats. Our results are the first to report the prevalence of *Eimeria* species based on molecular analysis.

## References

[1] Aarthi S, Dhinakar RG, Raman M, Gomathinayagam S, Kumanan K. 2010 Molecular prevalence and preponderance of *Eimeria* spp among chickens in Tamil Nadu India. Parasitology Research, 107(4), 1013–1017 20607286 10.1007/s00436-010-1971-2

[2] Abbas RZ, Iqbal Z, Sindhu ZD, Khan MN, Arshad M. 2008 Identification of cross-resistance and multiple resistance in *Eimeria tenella* field isolates to commonly used anticoccidials in Pakistan. Journal of Applied Poultry Research, 17, 361–368

[3] Al-Natour MQ, Suleiman MM, Abo-Shehada MN. 2002 Flock-level prevalence of *Eimeria* species among broiler chicks in northern Jordan. Preventive Veterinary Medicine, 53(4), 305–310 11937237 10.1016/s0167-5877(01)00281-1

[4] Awais MM, Akhtar M, Iqbal Z, Muhammad F, Anwar MI. 2012 Seasonal prevalence of coccidiosis in industrial broiler chickens in Faisalabad Punjab Pakistan. Tropical Animal Health and Production, 44(2), 323–328 22102015 10.1007/s11250-011-0024-x

[5] Chapman HD. 1976 Resistance of field isolates of *Eimeria* species to anticoccidial drugs. Avian Pathology, 5(4), 283–290 18777357 10.1080/03079457608418197

[6] Chapman HD. 1997 Biochemical, genetic and applied aspects of drug resistance in *Eimeria* parasites of the fowl. Avian Pathology, 26, 221–244 18483904 10.1080/03079459708419208

[7] Chapman HD. 1999 Anticoccidial drugs and their effects upon the development of immunity to *Eimeria* infections in poultry. Avian Pathology, 28, 521–535 16147561 10.1080/03079459994317

[8] Chapman HD, Shirley MW. 1989 Sensitivity of field isolates of *Eimeria* species to monensin and lasalocid in the chicken. Research in Veterinary Science, 46(1), 114–117 2922499

[9] Chapman HD, Johnson ZB. 1992 Oocysts of *Eimeria* in the litter of broilers reared to eight weeks of age before and after withdrawal of lasalocid or salinomycin. Poultry Science, 71(8), 1342–1347 10.3382/ps.0711342 1523182

[10] Centers for Disease Control and Prevention in Atlanta, Georgia, USA Epi Info Version 3.5.2. http://wwwn.cdc.gov/epiinfo/7/index.htm 12 17, 2010

[11] Dalloul RA, Lillehoj HS. 2006 Poultry coccidiosis: recent advancements in control measures and vaccine development. Expert Review of Vaccines, 5, 143–163 16451116 10.1586/14760584.5.1.143

[12] Fernandez S, Katsuyama AM, Kashiwabara AY, Madeira AM, Durham AM, Gruber A. 2004 Characterization of SCAR markers of *Eimeria* spp of domestic fowl and construction of a public relational database (The Eimeria SCARdb). FEMS Microbiology Letters, 238, 183–188 15336420 10.1016/j.femsle.2004.07.034

[13] Gasser RB, Woods WG, Wood JM, Ashdown L, Richards G, Whithear KG. 2001 Automated fluorescence-based approach for the specific diagnosis of chicken coccidiosis. Electrophoresis, 22, 3546–3550 11669540 10.1002/1522-2683(200109)22:16<3546::AID-ELPS3546>3.0.CO;2-8

[14] Hadipour MM, Olyaie A, Naderi M, Azad F, Nekouie O. 2011 Prevalence of *Eimeria* species in scavenging native chickens of Shiraz Iran. African Journal of Microbiology Research, 5(20), 3296–3299

[15] Haug A, Thebo P, Mattsson JG. 2007 A simplified protocol for molecular identification of *Eimeria* species in field samples. Veterinary Parasitology, 146(1–2), 35–45 17386979 10.1016/j.vetpar.2006.12.015

[16] Haug A, Gjevre AG, Skjerve E, Kaldhusdal M. 2008 A survey of the economic impact of subclinical *Eimeria* infections in broiler chickens in Norway. Avian Pathology, 37(3), 333–341 18568662 10.1080/03079450802050705

[17] Haug A, Gjevre AG, Thebo P, Mattsson JG, Kaldhusdal M. 2008 Coccidial infections in commercial broilers: epidemiological aspects and comparison of *Eimeria* species identification by morphometric and polymerase chain reaction techniques. Avian Pathology, 37(2), 161–170 18393094 10.1080/03079450801915130

[18] Hu J, Fuller L, McDougald LR. 2000 Do anticoccidials interfere with development of protective immunity against coccidiosis in broilers? Journal of Applied Poultry Research, 9(3), 352–358

[19] Iacob OC, Duma V. 2009 Clinical paraclinical and morphopathological aspects in cecal eimeriosis of broilers. Scientia Parasitologica, 10, 43–50

[20] Jeffers TK. 1974 Anticoccidial drug resistance: Differences between *Eimeria acervulina* and *E. tenella* strains within broiler houses. Poultry Science, 53, 1009–1013 10.3382/ps.0531009 4841687

[21] Kawazoe U, Fabio JD. 1994 Resistance to diclazuril in field isolates of *Eimeria* species obtained from commercial broiler flocks in Brazil. Avian Pathology, 23, 305–311 18671095 10.1080/03079459408418998

[22] Kiani R, Rasadi M, Mohammadian MN. 2007 Sources and routes of introduction of *Eimeria* oocysts into broiler chick’s houses. International Journal of Poultry Science, 6(12), 925–927

[23] Kučera J. 1990 Identification of *Eimeria* species in Czechoslovakia. Avian Pathology, 19, 59–66 18679914 10.1080/03079459008418656

[24] Lee BH, Kim WH, Jeong J, Yoo J, Kwon YK, Jung BY, Kwon JH, Lillehoj HS, Min W. 2010 Prevalence and cross-immunity of *Eimeria* species on Korean chicken farms. Journal of Veterinary Medical Science, 72(8), 985–989 20234110 10.1292/jvms.09-0517

[25] Lee KW, Lillehoj HS, Jang SI, Pagès M, Bautista DA, Pope CR, Ritter GD, Lillehoj EP, Neumann AP, Siragusa GR. 2012 Effects of in ovo vaccination and anticoccidials on the distribution of *Eimeria* spp in poultry litter and serum antibody titers against coccidia in broiler chickens raised on the used litters. Research in Veterinary Science, 93(1), 177–182 21641010 10.1016/j.rvsc.2011.05.005

[26] Lobago F, Worku N, Wossene A. 2005 Study on coccidiosis in Kombolcha poultry farm Ethiopia. Tropical Animal Health and Production, 37(3), 245–251 15747861 10.1023/b:trop.0000049302.72937.12

[27] Long PL, Joyner LP. 1984 Problems in the identification of species of *Eimeria*. Journal of Parasitology, 31(4), 535–541 10.1111/j.1550-7408.1984.tb05498.x 6392531

[28] Long PL, Millard BJ, Smith KM. 1979 The effect of some anticoccidial drugs on the development of immunity to coccidiosis in field and laboratory conditions. Avian Pathology, 8(4), 453–467 18770471 10.1080/03079457908418371

[29] Long PL, Johnson J, McKenzie ME, Perry E, Crane MS, Murray PK. 1986 Immunisation of young broiler chickens with low level infections of *Eimeria tenella, E. acervulina* or *E. maxima*. Avian Pathology, 15(2), 271–278 18766526 10.1080/03079458608436287

[30] Maes L, Vanparijs O, Marsboom R. 1991 Effect of diclazuril (Clinacox) on the development of protective immunity against *Eimeria tenella*: laboratory trial in broiler chickens. Poultry Science, 70(3), 504–508 10.3382/ps.0700504 2047343

[31] Morris GM, Gasser RB. 2006 Biotechnological advances in the diagnosis of avian coccidiosis and the analysis of genetic variation in *Eimeria*. Biotechnology Advances, 24(6), 590–603 16901674 10.1016/j.biotechadv.2006.06.001

[32] Morris GM, Woods WG, Richards DG, Gasser RB. 2007 Investigating a persistent coccidiosis problem on a commercial broiler-breeder farm utilising PCR-coupled capillary electrophoresis. Parasitology Research, 101(3), 583–589 17404757 10.1007/s00436-007-0516-9

[33] Munir K, Muneer MA, Tiwari A, Chaudhry RM, Muruganandan S. 2007 Effects of polyether ionophores on the protective immune responses of broiler chickens against Angara disease and Newcastle disease viruses. Veterinary Research Communications, 31(7), 909–929 17310330 10.1007/s11259-007-0030-7

[34] Ogedengbe JD, Hunter DB, Barta JR. 2011 Molecular identification of *Eimeria* species infecting market-age meat chickens in commercial flocks in Ontario. Veterinary Parasitology, 178(3–4), 350–354 21295915 10.1016/j.vetpar.2011.01.009

[35] Peek HW, Landman WJ. 2003 Resistance to anticoccidial drugs of Dutch avian *Eimeria* spp field isolates originating from 1996, 1999 and 2001. Avian Pathology, 32(4), 391–401 17585463 10.1080/0307945031000121149

[36] Peeters JE, Derijcke J, Verlinden M, Wyffels R. 1994 Sensitivity of avian *Eimeria* spp to seven chemical and five ionophore anticoccidials in five Belgian integrated broiler operations. Avian Diseases, 38, 483–493 7832701

[37] Qin ZR, Arakawa A, Baba E, Fukata T, Miyamoto T, Sasai K, Withanage GS. 1995 *Eimeria tenella* infection induces recrudescence of previous *Salmonella enteritidis* infection in chickens. Poultry Science, 74(11), 1786–1792 10.3382/ps.0741786 8614687

[38] Rose ME, Long PL. 1962 Immunity to four species of *Eimeria* in fowls. Immunology, 5, 79–92 14493839 PMC1424172

[39] Sasmal NK, Sinha PK, Senapati PK, Bhowmik MK. 1984 Acquired immunity to *Eimeria tenella* in lasalocid-treated chickens. Veterinary Parasitology, 15(1), 1–9 6541392 10.1016/0304-4017(84)90105-5

[40] Schnitzler BE, Thebo PL, Mattsson JG, Tomley FM, Shirley MW. 1998 Development of a diagnostic PCR assay for the detection and discrimination of four pathogenic *Eimeria* species of the chicken. Avian Pathology, 27, 490–497 18484033 10.1080/03079459808419373

[41] Schnitzler BE, Thebo PL, Tomley FM, Uggla A, Shirley MW. 1999 PCR identification of chicken *Eimeria*: a simplified read-out. Avian Pathology, 28(1), 89–93 16147553 10.1080/03079459995091

[42] Shalaby MA, El-Sanousi AA, Yehia MM, Naser A, Reda IM. 1993 The effect of salinomycin on the immune response of chicks. Deutsche tierarztliche Wochenschrift, 100(5), 182–185 8319544

[43] Shirley MV. 1995 *Eimeria* species and strains, in Guidelines on Techniques in Coccidiosis, Eckert J, Braun R, Shirley MW, Coudert P, (Eds.) COST89/820 Biotechnology, Research European Commission, Luxembourg, p. 1–25

[44] Shirzad MR, Seifi S, Gheisari HR, Hachesoo BA, Habibi H, Bujmehrani H. 2011 Prevalence and risk factors for subclinical coccidiosis in broiler chicken farms in Mazandaran province Iran. Tropical Animal Health and Production, 43(8), 1601–1604 21626064 10.1007/s11250-011-9876-3

[45] Stephen B, Rommel M, Daugschies A, Haberkorn A. 1997 Studies of resistance to anticoccidials in *Eimeria* field isolates and pure *Eimeria* strains. Veterinary Parasitology, 69, 19–29 9187026 10.1016/s0304-4017(96)01096-5

[46] Stucki U, Braun R, Roditi I. 1993 *Eimeria tenella*: characterization of a 5S ribosomal RNA repeat unit and its use as a species-specific probe. Experimental Parasitology, 76, 68–75 8467900 10.1006/expr.1993.1008

[47] Sun XM, Pang M, Jia T, Yan WC, He G, Hao LL, Bentue M, Sue X. 2009 Prevalence of *Eimeria* species in broilers with subclinical signs from fifty farms. Avian Diseases, 53, 301–305 19630240 10.1637/8379-061708-Resnote.1

[48] Tsuji N, Kawazu S, Ohta M, Kamio T, Isobe T, Shimura K, Fujisaki K. 1997 Discrimination of eight chicken *Eimeria* species using the two-step polymerase chain reaction. Journal of Parasitology, 83, 966–970 9379312

[49] Uniunea crescatorilor de pasari din Romania (Romanian Association of Poultry Breeders) 2013 http://www.avicultura.ro/, consulted on November 27, 2012

[50] Williams RB. 1973 The effect of *Eimeria acervulina* on the reproductive potentials of four other species of chicken coccidia during concurrent infections. British Veterinary Journal, 129(3), xxix–xxxi 4738008 10.1016/s0007-1935(17)36498-9

[51] Williams RB. 2001 Quantification of the crowding effect during infections with the seven *Eimeria* species of the domesticated fowl: its importance for experimental designs and the production of oocyst stocks. International Journal of Parasitology, 31(10), 1056–1069 11429169 10.1016/s0020-7519(01)00235-1

[52] Williams RB. 2005 Intercurrent coccidiosis and necrotic enteritis of chickens: rational integrated disease management by maintenance of gut integrity. Avian Pathology, 34(3), 159–180 16191699 10.1080/03079450500112195

[53] Williams RB. 2006 Tracing the emergence of drug-resistance in coccidia (*Eimeria* spp) of commercial broiler flocks medicated with decoquinate for the first time in the United Kingdom. Veterinary Parasitology, 135, 1–14 16289564 10.1016/j.vetpar.2005.10.012

[54] Williams RB, Bushell AC, Reperant JM, Doy TG, Morgan JH, Shirley MW, Yvore P, Carr MM, Fremont Y. 1996 A survey of *Eimeria* species in commercially-reared chickens in France during 1994. Avian Pathology, 25(1), 113–130 18645842 10.1080/03079459608419125

[55] Williams RB, Marshall RN, La Ragione RM, Catchpole J. 2003 A new method for the experimental production of necrotic enteritis and its use for studies on the relationships between necrotic enteritis coccidiosis and anticoccidial vaccination of chickens. Parasitology Research, 90(1), 19–26 12743800 10.1007/s00436-002-0803-4

[56] Williams RB, Marshall RN, Pagés M, Dardi M, del Cacho E. 2009 Pathogenesis of *Eimeria praecox* in chickens: virulence of field strains compared with laboratory strains of *E. praecox* and *Eimeria acervulina*. Avian Pathology, 38(5), 359–366 19937523 10.1080/03079450903186028

[57] Zhang JJ, Wang LX, Ruan WK, An J. 2013 Investigation into the prevalence of coccidiosis and maduramycin drug resistance in chickens in China. Veterinary Parasitology, 191, 29–34 22925822 10.1016/j.vetpar.2012.07.027

